# Knockdown of CRAD suppresses the growth and promotes the apoptosis of human lung cancer cells via Claudin 4

**DOI:** 10.1042/BSR20201140

**Published:** 2020-10-13

**Authors:** Anfang Cui, Yuchan Xue, Xi’ao Wang, Yanhong Huang, Xiaolin Han, Xiangling Li, Delei Niu, Shaorui Niu, Yujie Zhao, Xinyu Yang, Wei Yu

**Affiliations:** 1 College of Basic Medicine, Jining Medical University, Jining, Shandong 272067, P.R. China; 2 Institute of Forensic Medicine and Laboratory Medicine, Jining Medical University, Jining, Shandong 272067, P.R. China

**Keywords:** Apoptosis, CARD, Claudin 4, NSCLC, Proliferation

## Abstract

Non–small cell lung cancer (NSCLC) is one of the most common causes of cancer-related mortality globally. However, the mechanism underlying NSCLC is not fully understood. Here, we investigated the role of cancer-related regulator of actin dynamics (CRAD) in NSCLC. We showed that CRAD was up-regulated in human NSCLC tissues and lung cancer cell lines. Lentivirus-mediated knockdown of CRAD repressed the proliferation and colony growth of A549 and H1299 cells. Apoptosis was enhanced by CRAD silencing in both cells, implicating that CRAD might maintain the survival of lung cancer cells. Microarray and bioinformatic assay revealed that CRAD directly or indirectly regulated diverse genes, including those involved in cell cycle and DNA damage repair. qRT-PCR and Western blot results confirmed the dysregulated genes as shown in microarray analysis. Claudin 4 was up-regulated in CRAD silenced A549 cells. The knockdown of Claudin 4 blocked the effects of CRAD on the expression of cell cycle and apoptosis effectors and enhanced the viability of A549 cells with CRAD down-regulation. Taken together, our findings demonstrate that CRAD acts as an oncogene in NSCLC at least partly through repressing Claudin 4.

## Introduction

Lung cancer is a serious public health problem and it remains the most common cause of cancer-related mortality worldwide [1]. Non–small cell lung cancer (NSCLC), which comprises three histological subtypes (adenocarcinoma, squamous cell carcinoma, and large cell carcinoma), accounts for approximately 80% of all cases of lung cancers [2]. Advanced molecular screening has directed the use of targeted therapy in patients with advanced-stage of NSCLC [3]. However, the mechanism underlying human lung cancer is not fully understood and the prognosis is still poor [4]. A better insight into the cellular and molecular mechanism underlying human lung cancer would facilitate the development of therapeutic strategies for human lung cancer [5].

Actin dynamics plays a critical role in various biological processes, including cell migration, development, tissue remodeling, and memory formation [6,7]. Actin dynamics and competition for myosin monomer govern the sequential amplification of myosin filaments [8,9]. During would healing, circadian actin dynamics drives rhythmic fibroblast mobilization [10]. Actin dynamics provides membrane tension to merge fusing vesicles into the plasma membrane [11]. The dynamics of the cytoskeletal actin is regulated by diverse factors, and most of these regulators participate in the development of cancer [12].

Cancer-related regulator of actin dynamics (CRAD) is an actin-binding cytoskeletal protein [13]. CRAD is expressed within several early neuronal tissues destined to give rise to central, peripheral, and sympathetic nervous system structures [14]. CRAD regulates Wnt signaling by increasing actin polymerization and stabilizing AJs in the intestinal epithelium [15]. In mice, Crad knockout induces the loss of epithelial cell integrity and activates Wnt signaling, which leads to the development of intestinal mucinous adenoma. In the colorectum, with APC mutation, Crad knockout initiates and accelerates mucinous and invasive adenoma development with APC mutation [15]. However, the role of CRAD in other cancer is not known.

In the present study, we aimed to investigate the function of CRAD in human lung cancer. We found that the expression of CRAD was overexpressed in human NSCLC tissues and cell lines. Lentivirus-mediated knockdown of CRAD in lung cancer cells repressed proliferation, reduced colony formation and induced apoptosis of the cells. Microarray and bioinformatic analysis revealed that CRAD regulated diverse genes involved in cell cycle and DNA damage repair.

## Materials and methods

### Clinical data analysis

The expression of CRAD in normal lung tissues (59) and lung cancer tissues (*n*=526), as well as 57 matched cancer versus adjacent lung tissues, were analyzed using The Cancer Genome Atlas (TCGA) databases. The 57 matched cancer cases are involved in the 59 normal lung tissues.

### Quantitative real-time PCR

TRIzol reagent (ThermoFisher, #10296010) was applied to extract total RNA from cultured cells. Then cDNA was synthesized from 1 μg of total RNA with the PrimeScript™ 1st strand cDNA Synthesis Kit (TaKaRa, #6110A). Next, quantitative real-time PCR (qRT-PCR) was carried out to analyze the relative mRNA level of target genes by using SYBR® Premix Ex Taq™ (TaKaRa, #RR420A). The primers used for qRT-PCR are listed below:

GAPDH forward: 5′-TGTGGGCATCAATGGATTTGG-3′
GAPDH reverse: 5′- ACACCATGTATTCCGGGTCAAT-3′
CRAD forward: 5′-AGACCTCCAAACAGAGCACG-3′
CRAD reverse: 5′- GGGTAAGGAAGGCTTGGGAC-3′
Claudin 4 forward: 5′-CGGCCCACAACATCATCCAA-3′
Claudin 4 reverse: 5′-GGCGGAGTAAGGCTTGTCT-3′
CASP1 forward: 5′-TTTCCGCAAGGTTCGATTTTCA-3′
CASP1 reverse: 5′-GGCATCTGCGCTCTACCATC-3′
CASP4 forward: 5′-CAAGAGAAGCAACGTATGGCA-3′
CASP4 reverse: 5′-AGGCAGATGGTCAAACTCTGTA-3′
CDKN1A forward: 5′-TGTCCGTCAGAACCCATGC-3′
CDKN1A reverse: 5′-AAAGTCGAAGTTCCATCGCTC-3′
CDK1 forward: 5′-AAACTACAGGTCAAGTGGTAGCC-3′
CDK1 reverse: 5′-TCCTGCATAAGCACATCCTGA-3′
CCNB1 forward: 5′-AATAAGGCGAAGATCAACATGGC-3′
CCNB1 reverse: 5′-TTTGTTACCAATGTCCCCAAGAG-3′
CCNB2 forward: 5′-TGCTCTGCAAAATCGAGGACA-3′
CCNB2 reverse: 5′-GCCAATCCACTAGGATGGCA-3′

### Western blot

Freshly cultured cells were subjected to protein extraction with RIPA (Beyotime, #P0013B) supplied with a proteinase inhibitor cocktail (Promega, #G6521). Western blot was performed as described previously [16,17]. The following primary antibodies were used: Anti-CRAD antibody (Sigma, #HPA043249) and anti-GAPDH antibody (Santa Cruz Biotechnology, #sc-32233).

### Cell lines and cell culture

HEK293FT cells, 95D cells, H1299 cells, H1975 cells, BEAS-2B and A549 cells were purchased from ATCC. The cells were cultured in high glucose-containing DMEM (ThermoFisher, #12491-015) supplemented with 10% fetal bovine serum (ThermoFisher, #10099133), 100 units/ml penicillin, and 100 µg/ml streptomycin. All cells were cultured at 37°C under a humidified atmosphere containing 5% CO_2._

### Lentivirus packaging and transduction

The lentivirus was packaged with the ViraPower™ II Lentiviral Gateway® Expression System (ThermoFisher, #K36720). The shRNA sequence targeting GRAD and Claudin 4 is 5′-CCGCCAGTCAATGCAAAGTTCTCTA-3′ and 5′-TATGGTGATAGTGCCGGTGTC-3′, respectively. The oligos were synthesized by Invitrogen. For lentivirus packaging, HEK293FT cells were co-transfected with the lentiviral particles as described previously [18,19]. For transduction, A549 and H1299 cells were incubated with virus-containing supernatant with 4 μg/ml polybrene (Sigma, #107689). Forty-eight hours later, the infected cells were then selected for an additional 72 h with puromycin (5 μg/ml).

### Cell proliferation assay

MTT (3-(4,5-dimethylthiazol-2-yl)-2,5-diphenyltetrazolium bromide) assay was performed in A549 and H1299 cells using MTT Cell Proliferation and Cytotoxicity Assay Kit (BOSTER, # AR1156) according to the manufacturer’s protocol.

### Cell colony formation assay

Soft sugar colony formation assay was performed using A549 and H1299 cells with/without CRAD knockdown as described previously [20,21]. A549 and H1299 cells were cultured for 14 days and cell colonies were stained with 0.005% Crystal Violet and analyzed using a microscope. The colony number in each well was calculated.

### FACS-based apoptosis analysis

FACS was performed as described previously [22,23]. The apoptosis of A549 and H1299 cells was analyzed by staining with Annexin V-APC (Bioscience) according to the manufacturer’s protocol. For analysis of apoptosis, cells were harvested after incubation for 4 days, washed with PBS, and resuspended using a staining buffer at a final density of 1–10 × 10^6^/ml. Then, 5 μl annexin V-APC was added into 100 μl of the above cell suspensions and incubated at room temperature for 15 min and subjected to flow cytometry analysis.

### Microarray and bioinformatic analysis

Total RNA from A549 cells was extracted using Trizol reagent. Affymetrix human GeneChipprimeview was used for microarray processing to determine gene expression profiles according to the manufacturer’s instructions. Significantly different genes between A549 cells with/without CRAD knockdown were identified depending on the following criteria: *P*<0.05 and the absolute fold change >1.5. Pathway enrichment analysis was performed for all significantly different genes based on KEGG and Reactome Pathway Database databases with Gene Set Enrichment Analysis.

### Statistical analysis

All the values are expressed and mean ± SEM of at least three independent experiments if no other statement is provided. The difference between the two groups was analyzed by paired or unpaired Student’s *t*-test. One-way ANOVA analysis followed by Bonferroni post-hoc test was applied for analyzing the difference between groups more than three when the data matched the standard. All the statistical analysis was carried out by using the GraphPad Prism 7.0. *P*<0.05 was considered significant.

## Results

### CRAD expression is overexpressed in human lung cancer tissues and cell lines

**Figure 1:**
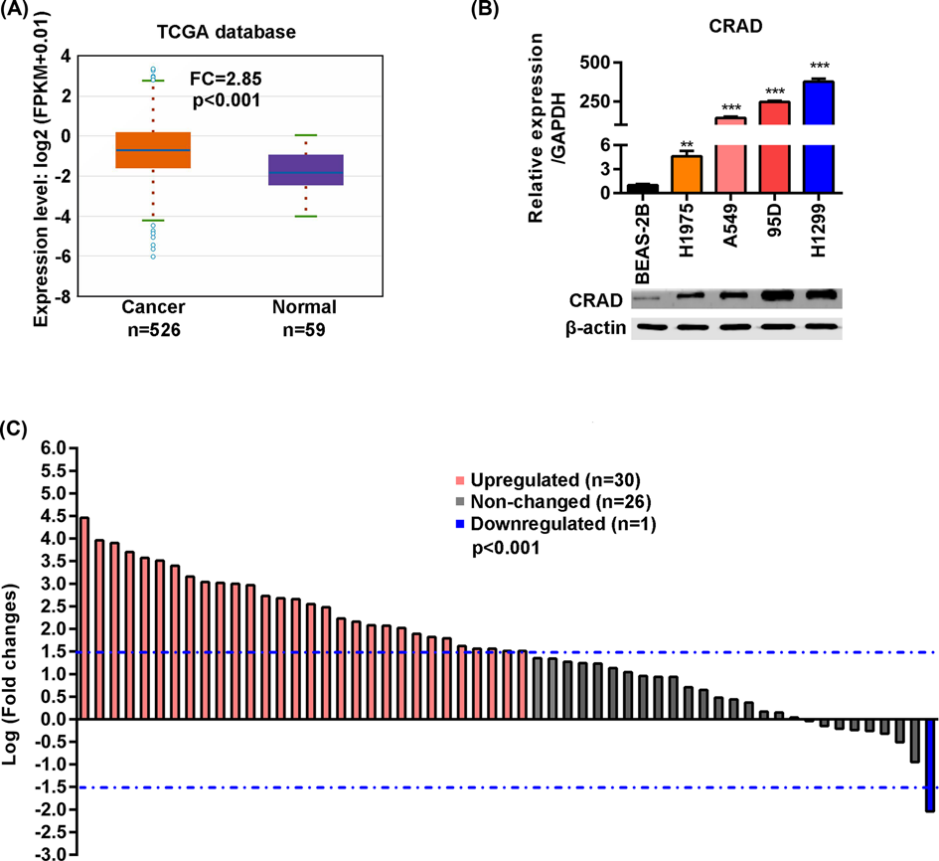
CRAD is overexpressed in human lung cancer (**A**) CRAD mRNA level is higher in lung cancer tissues compared with normal control tissues. The analysis was based on the data from the TCGA database; *n*=59 in the control group, *n*=526 in cancer group, *P*<0.001 by Student’s *t* test. (**B**) Fold change of CRAD mRNA in human non–small cell lung cancer compared with adjacent normal tissues; *n*=57, *P*<0.001. (**C**) CRAD mRNA and protein levels are higher in lung cancer cells than epithelial cells from the normal human bronchial epithelium. The mRNA and protein from lung cancer cells (95D, H1299, H1975, and A549) and normal epithelial cells (BEAS-2B) were subjected to qRT-PCR and Western blot analysis, respectively; **P<0.01, ***P<0.001 versus BEAS-2B by one-way ANOVA followed by Bonferroni post-hoc test.

The potential function of CRAD in human lung cancer remains unknown. To explore the roles of CRAD protein in human lung cancer and the underlying mechanism, we first analyzed the expression of CRAD in human lung cancer with the TCGA database. The results showed that CRAD expression in human lung cancer was significantly up-regulated (Figure 1A). Next, we analyzed the expression of CRAD with information of 57 lung tissues and adjacent normal tissues from the TGCA database. Within 84% of these patients, the level of CRAD was up-regulated in cancer tissues compared with the non-cancer adjacent tissues (Figure 1B). Next, the mRNA and protein levels of CRAD in lung cancer cells (95D, H1299, H1975, and A549) and normal epithelial cells (BEAS-2B) were analyzed by qRT-PCR and Western blot. The results showed that the mRNA and protein of CRAD were significantly higher in lung cancer cells than in normal epithelial cells (Figure 1C). Taken together, these findings demonstrated that the expression of CRAD was up-regulated in human lung cancer tissues and cell lines.

### The knockdown of CRAD represses the growth of lung cancer cells

**Figure 2:**
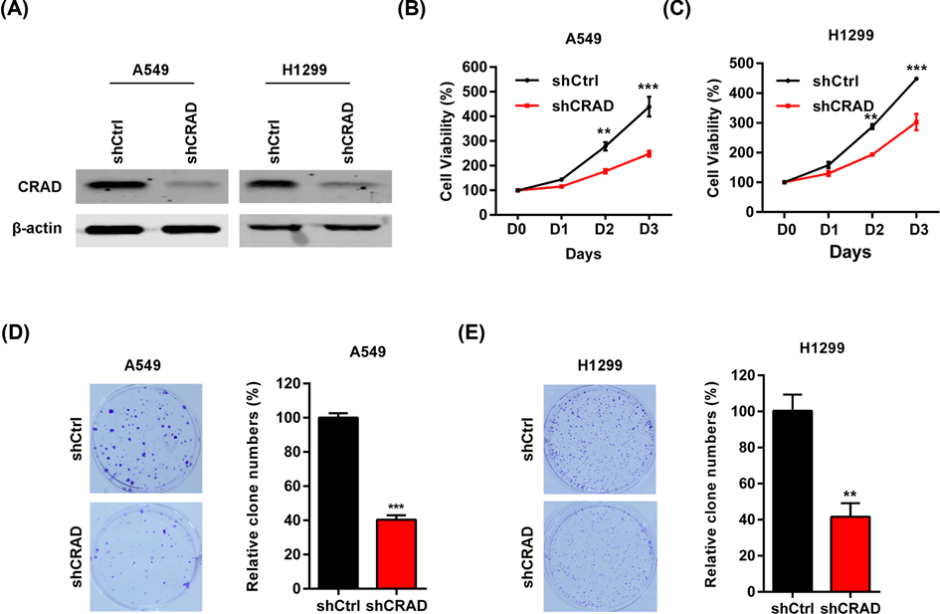
CRAD knockdown represses the proliferation rate of lung cancer cells (**A**) Representative Western blot results showing CRAD knockdown in lung cancer cells. Lung cancer A549 and H1299 cells were infected with lentivirus carrying shCRAD or control shRNA (shCtrl) for 48 h. The protein was subjected to Western blot analysis. (**B**) CRAD knockdown reduces the cell proliferation rate of A549 lung cancer cells. Selected A549 cells with/without CRAD knockdown were cultured and the cell count was evaluated every day for 4 days. **P<0.01 and ***P<0.001 versus shCtrl by one-way ANOVA followed by Bonferroni post-hoc test. (**C**) CRAD knockdown reduces the cell proliferation rate of H1299 lung cancer cells. Selected H1299 cells with/without CRAD knockdown were cultured and the cell count was evaluated every day for 4 days. **P<0.01 and ***P<0.001 versus shCtrl by one-way ANOVA followed by Bonferroni post-hoc test. (**D**) CRAD knockdown inhibits colony formation of A549 lung cancer cells. Selected A549 cells with/without CRAD knockdown were used for colony formation assay. The cells were cultured for 14 days. ***P<0.001 versus shCtrl by Student’s *t* test. (**E**) CRAD knockdown inhibits colony formation of H1299 lung cancer cells. Selected H1299 cells with/without CRAD knockdown were used for colony formation assay. The cells were cultured for 14 days. **P<0.01 versus shCtrl by Student's *t* test.

The overexpression of CRAD in human lung cancer tissues and cell lines implicated the potential function of CRAD in human lung cancer. To explore the roles of CRAD in human lung cancer cells, we performed lentivirus to express shRNA targeting CRAD (shCRAD). Lung cancer A549 and H1299 cells were infected with lentivirus carrying shCRAD, the expression of CRAD was analyzed by Western blot. The results demonstrated that the protein levels of CRAD were significantly knocked down in A549 and H1299 cells by the shCRAD (Figure 2A). Selected A549 and H1299 cells with/without CRAD knockdown were subjected to cell proliferation assay. MTT assay revealed that the proliferation of A549 and H1299 cells were significantly repressed since day 3 by CRAD knockdown (Figure 2B,C). Therefore, these results demonstrated that CRAD knockdown represses the proliferation of lung cancer cells.

### The knockdown of CRAD inhibits the colony formation capacity of lung cancer cells

Self-renew or colony formation is a feature of cancer cells [24,25]. We next investigated the effects of CRAD knockdown on the colony formation of lung cancer cells. Selected A549 and H1299 cells with/without CRAD knockdown were subjected to colony formation assay. The cells were cultured for 2 weeks, and then the colony number was analyzed. The results showed that the colony number of A549 and H1299 cells was reduced by CRAD knockdown (Figure 2D,E). Therefore, CRAD overexpression might be a potential reason for the high colony formation capacity of A549 and H1299 cells.

### Knockdown of CRAD induces apoptosis of lung cancer cells

**Figure 3:**
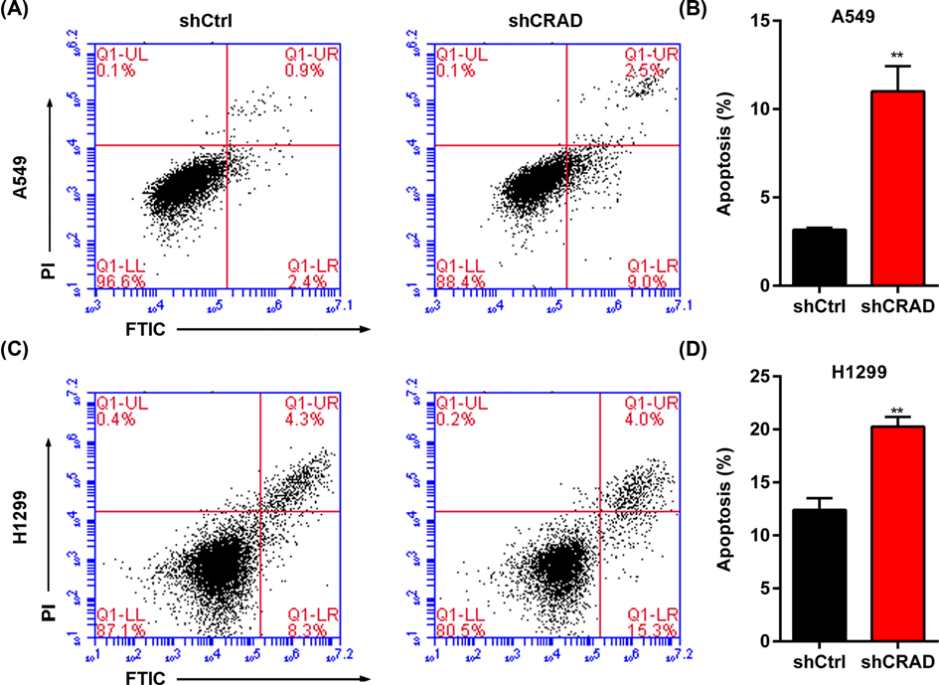
CRAD knockdown induces apoptosis of lung cancer cells (**A**) Representative FACS results showing CRAD knockdown induces apoptosis of A549 lung cancer cells. Selected A549 cells with/without CRAD knockdown were cultured for 4 days and the cell apoptosis was analyzed with FACS. (**B**) Quantitative results showing CRAD knockdown increases the percentage of apoptotic lung cancer cells in A549 cells. Selected A549 cells with/without CRAD knockdown were cultured for 4 days and the cell apoptosis was analyzed with FACS. **P<0.01 versus shCtrl by Student's *t* test. (**C**) Representative FACS results showing CRAD knockdown induces apoptosis of H1299 lung cancer cells. Selected H1299 cells with/without CRAD knockdown were cultured for 4 days and the cell apoptosis was analyzed with FACS. (**D**) Quantitative results showing CRAD knockdown increases the percentage of apoptotic lung cancer cells in H1299 cells. Selected H1229 cells with/without CRAD knockdown were cultured for 4 days and the cell apoptosis was analyzed with FACS. **P<0.01 versus shCtrl by Student's *t* test.

Low basic apoptosis or high anti-apoptotic ability a common hallmark of lung cancer cells [26]. To investigate whether CRAD regulates the survival or apoptosis of lung cancer cells, we selected A549 cells with/without CRAD knockdown. The selected cells were cultured for 4 days and then the cell apoptosis was analyzed by FACS assay. The results demonstrated that CRAD knockdown promoted the apoptosis of A549 cells (Figure 3A,B). The role of CRAD in regulating apoptosis was also observed in H1299 cells (Figure 3C,D). Therefore, CRAD regulates the survival or anti-apoptotic capacity of lung cancer cells.

### Microarray-based analysis of CRAD downstream genes

**Figure 4:**
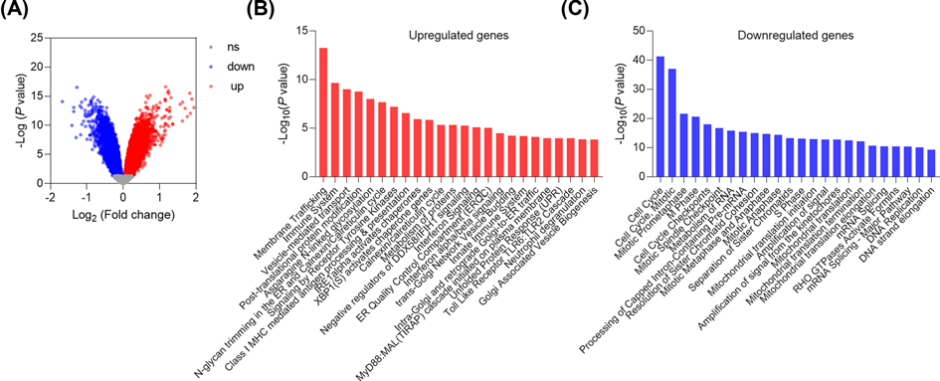
Microarray analysis of CRAD targets (**A**) Results showing 1963 genes (861 genes down-regulated and 1102 genes up-regulated) with differential expressions. Selected A549 cells with/without CRAD knockdown were subjected to microarray assay (criteria: *P*<0.05). (**B**) Functional pathway enrichment of up-regulated genes by CRAD knockdown was analyzed based on KEGG. The enrichment analysis was performed with the g: Profiler (https://biit.cs.ut.ee/gprofiler/gost). (**C**) Functional pathway enrichment of downregulated genes by CRAD knockdown was analyzed based on KEGG. The enrichment analysis was performed with the g: Profiler (https://biit.cs.ut.ee/gprofiler/gost).

**Figure 5:**
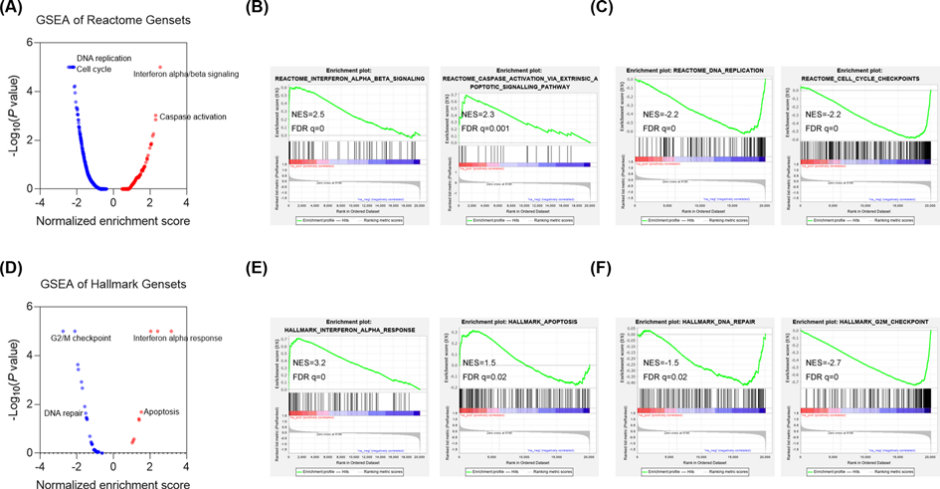
Gene set enrichment analysis of CRAD targets (**A**) GSEA of the total 1499 Reactome gene sets. (**B**) GSEA showing enrichment of the Reactome gene sets of Interferon alpha/beta responses and signaling and Caspase activation in the shCRAD group. (**C**) GSEA showing the enrichment of the Reactome gene sets of DNA replication and cell cycle checkpoint in the shCtrl group. (**D**) GSEA of 50 Hallmarks gene sets. (**E**) GSEA showing enrichment of hallmark gene sets interferon-α response and apoptosis in the shCRAD group. (**F**) GSEA showing enrichment of hallmark gene sets DNA repair and G2/M checkpoint in shCtrl group.

We then performed a microarray analysis to further investigate the mechanism underlying CRAD function in lung cancer cells. The microarray data showed that 861 genes were down-regulated whereas 1102 genes were up-regulated by CRAD knockdown in A549 cells. (Figure 4A). Our functional pathway enrichment of differentially expressed genes was analyzed based on the Kyoto Encyclopedia of Genes and Genomes (KEGG) databases and the results showed that several pathways involved in diverse types of cancer were regulated by CRAD. Significantly, the interferon signaling was activated by CRAD knockdown whereas the cell cycle pathway was repressed by CRAD knockdown (Figure 4B,C). Furthermore, we performed GSEA of the Reactome Pathway, the results revealed the enrichment of interferon and apoptosis gene sets and repression of DNA replication and cell cycle gene sets in lung cancer cells with CRAD knockdown (Figure 5A–C). Finally, we analyzed the enrichment of hallmark gene sets. The results showed the enrichment of interferon and apoptosis gene sets and repression of the G2/M checkpoint and DNA repair pathway in lung cancer cells with CRAD knockdown (Figure 5D–F).

### CRAD knockdown reduces cell viability partly through up-regulating Claudin 4

**Figure 6:**
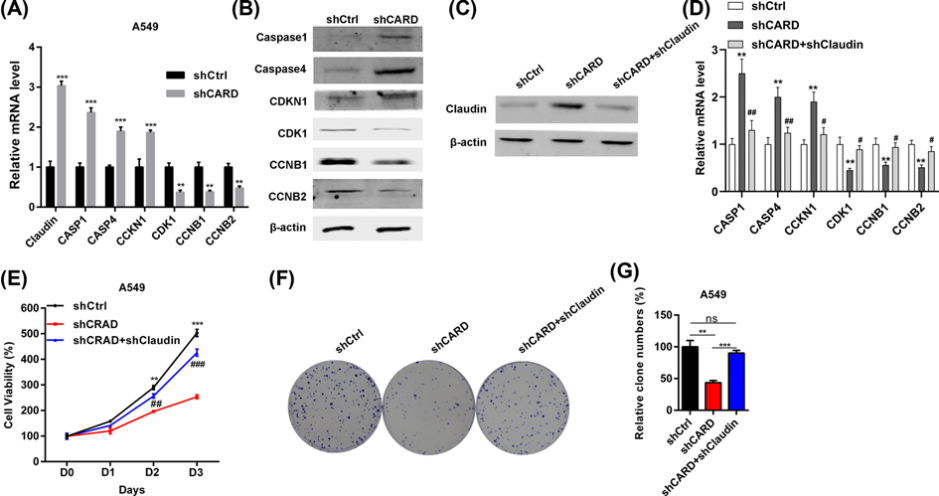
Knockdown of Claudin 4 increases the proliferation of A549 cells with down-regulation of CRAD (**A**) qRT-PCR results of Claudin 4, CASP1, CASP4, CCKN1, CDK1, CCNB1, and CCNB2 in shCtrl and shCRAD A549 cells; **P<0.01, ***P<0.001 by Student's *t* test. (**B**) Western blot results of CASP1, CASP4, CCKN1, CDK1, CCNB1 and CCNB2 in shCtrl and shCRAD A549 cells. (**C**) Western blot results of Claudin 4 in shCtrl, shCRAD and shCRAD+shClaudin 4 A549 cells. (**D**) qPCR results showing knockdown of Claudin 4 blocked CRAD effects on the expression of cell cycle and apoptosis effectors; **P<0.01 versus shCtrl; #*P*<0.05, ##*P*<0.01 versus shCRAD by one-way ANOVA followed by Bonferroni *post-hoc* test. (**E**) shCtrl, shCRAD and shCRAD+shClaudin 4 A549 cells were subjected to CCK8 analysis of proliferation. ** and ##, *P*<0.01. *** and ###, *P*<0.001 by one-way ANOVA followed by Bonferroni *post-hoc* test. (**F** and **G**) shCtrl, shCRAD and shCRAD+shClaudin 4 A549 cells were subjected to colony growth assay. ** and ##, *P*<0.01. *** and ###, *P*<0.001 by one-way ANOVA followed by Bonferroni post-hoc test.

Finally, qRT-PCR and Western blot experiments were performed to confirm the dysregulated genes as shown in microarray analysis. The results showed that CASP1, CASP4, and CCKN1 were up-regulated in CRAD knockdown A549 cells (Figure 6A,B). By contrast, CDK1, CCNB1, and CCNB2 were down-regulated after CRAD knockdown in A549 cells (Figure 6A,B). We also found that Claudin 4 was up-regulated in CRAD silenced A549 cells (Figure 6A,C). Previous studies showed that Claudin 4 is a pivotal upstream regulator of apoptosis and cell cycle [27,28]. We then explored the role of up-regulated Claudin 4 in A549 cells by further knocking down Claudin 4. Western blot results showed that up-regulated Claudin 4 was efficiently silenced in A549 cells (Figure 6C). Besides, Claudin 4 knockdown blocked the effects of CRAD on the expression of cell cycle and apoptosis effectors (Figure 6D). The decreased proliferation and colony formation was enhanced by Claudin 4 silencing in A549 cells with down-regulation of CRAD (Figure 6E–G). These results suggest that CRAD knockdown represses the viability of A549 cells at least partly through up-regulating Claudin 4.

## Discussion

In this work, we identified CRAD as an oncogenic factor in human NSCLC. The expression of CRAD was up-regulated in human NSCLC tissues and cell lines. Our lentivirus-mediated loss-of-function experiments demonstrated that CRAD knockdown repressed the proliferation and colony formation capacity of lung cancer cells. In addition, CRAD knockdown induces apoptosis of lung cancer cells. Our microarray and bioinformatic analysis showed that CRAD regulated cell cycle and DNA damage in lung cancer cells, which may account for the function of CRAD in human lung cancer.

CRAD is an actin-binding protein. CARD regulates Wnt signaling by increasing actin polymerization [15]. Previous studies showed that Claudin 4 is a pivotal upstream regulator of apoptosis and cell cycle [27,28]. To investigate the cellular functions of CRAD in human NSCLC cells, we generated lentivirus carrying shRNA that targets CRAD in A549 and H1299 cells. Indeed, our real-time PCR demonstrated that knockdown of Claudin 4 repressed the effects of CRAD on these apoptosis and cell cycle regulators. Several pieces of proliferation assays demonstrated that CRAD knockdown repressed the proliferation rate of lung cancer cells. Furthermore, colony formation was also performed to study the roles of CRAD. We found that CRAD knockdown reduced the capacity of colony formation of lung cancer cells. Interestingly, a previous work defined CRAD as a tumor suppressor, of which inactivation deregulates the cytoskeleton and hyperactivates Wnt signaling, initiating mucinous colorectal cancer [15]. These opposing results may be due to the different cell lines and the effects of CRAD on cancer cells may be not direct.

We also observed that CRAD knockdown led to apoptosis of lung cancer cells, which may underline the effects of CRAD on the proliferation and colony formation of lung cancer cells. Therefore, the CRAD family members maintain the survival of cells. Importantly, we carried out microarray and bioinformatic experiments and confirmed that CRAD regulated cell cycle and DNA damage repair in lung cancer cells. Therefore, CRAD regulated neither cell cycle nor DNA damage repair to maintain the survival, high proliferation rate and colony formation capacity of lung cancer cells.

The roles of interferon signaling in human lung cancer have been widely studied [29]. For instance, interferon-γ (IFN-γ)-mediated inhibition of lung cancer is correlated with PD-L1 expression and is regulated by the PI3K-AKT signaling [30]. Tumor-derived extracellular vesicles “educate” healthy cells to promote metastases. It was reported that interferon drove oxysterol to defense against tumor-derived extracellular vesicles [31]. Interestingly, we observed that CRAD knockdown activated the Interferon signaling. Further study is needed to investigate how CRAD regulates interferon response and whether this role of CRAD contributes to its function in lung cancer.

Claudins (CLDNs) belongs to the family of tight-junction proteins that regulate the polarity and differentiation of different cells. As a member of the CLDNs family, CLDN4 has been shown to participate in cancer development. CLDN4 is related to the tumor progression and malignancy of gastric cancer [32]. The silencing of CLDN4 activates PI3K/AKT signaling pathway and potentiates the proliferation of gastric cancer cells [33]. However, the upstream regulator of CLDN4 in cancers remains unknown. Here, we identified CLDN4 as the downstream effector of CRAD. Knockdown of CRAD up-regulated CLDN4 in A549 cells. Interfering CLDN4 in CRAD silenced A549 cells led to enhanced proliferation and colony growth of the cells. These results revealed CRAD as the upstream modulator of CLDN4 in lung cancer cells.

## Conclusions

In conclusion, our findings demonstrate that CRAD is overexpressed in human NSCLC tissues and promotes the survival, proliferation and colony formation of lung cancer cells. Therefore, CRAD may be a potential target for the treatment of human NSCLC.

## Data Availability

The data used to support the findings of this study are available from the corresponding author upon request.

## Abbreviations

CLDN: Claudin
CRAD: cancer-related regulator of actin dynamics
MTT: 3-(4,5-dimethylthiazol-2-yl)-2,5-diphenyltetrazolium bromide
NSCLC: non–small cell lung cancer
